# Comparison of a traditional bovine respiratory disease control regimen with a targeted program based upon individualized risk predictions generated by the Whisper On Arrival technology

**DOI:** 10.1093/tas/txab081

**Published:** 2021-05-06

**Authors:** Jason S Nickell, John P Hutcheson, David G Renter, David A Amrine

**Affiliations:** 1 Allflex Livestock Intelligence, A Subsidiary of Merck Animal Health, Madison, WI 53718; 2 Merck Animal Health, De Soto, KS 66018; 3 Center for Outcomes Research and Epidemiology, Kansas State University, Manhattan, KS 66506; 4 Beef Cattle Institute, Kansas State University, Manhattan, KS 66506

**Keywords:** bovine respiratory disease complex, cattle, feedlot, metaphylaxis, Whisper

## Abstract

The study objective was to determine if cattle health and performance comparing a targeted bovine respiratory disease (BRD) control program based on individualized risk prediction generated by a novel technology (Whisper On Arrival) was superior to a negative control (no metaphylaxis) yet no different than a positive control (conventional BRD control; 100% application). Across four study sites, auction market-derived beef calves were randomly allocated to one of four BRD control treatment groups: 1) Negative control (Saline), 2) Positive control (Tildipirosin [TIL] to 100% of the group), 3) Whisper-high (±TIL based on conservative algorithm threshold), and 4) Whisper-low (±TIL based on aggressive algorithm threshold). Within either Whisper On Arrival group, only calves predicted to be above the algorithm threshold by the technology (determined a priori) were administered TIL leaving the remainder untreated. Cattle were followed to either a short-term timepoint (50 or 60 d; health outcomes, all sites; feed performance outcomes, two sites) or to closeout (two sites). Data were analyzed as a completely randomized block design separately at each site. Across all sites, BRD control antibiotic use was reduced by 11% to 43% between the two Whisper On Arrival treatment groups compared to the positive control. The positive control and both Whisper On Arrival groups reduced (*P* ≤ 0.05) BRD morbidity compared to negative controls at both the short-term timepoint at three of the four sites and at closeout at one of two sites. The positive control and both Whisper-managed groups had improved (*P* ≤ 0.05) average daily gain (ADG), dry-matter intake (DMI), and feed efficiency compared to negative controls at the short-term timepoint at one of two sites. At closeout, the positive control and both Whisper-managed groups improved (*P* ≤ 0.05) ADG (deads-in) compared to the negative control at one of the two sites. At one of two sites, the positive control and the Whisper-high group displayed an improvement (*P* ≤ 0.05) in hot carcass weight compared to the negative control. The Whisper On Arrival technology maintained the benefits of a conventional BRD control program yet reduced BRD control antibiotic use by 11% to 43%. This technology maintained the benefits of a conventional BRD control program while reducing antibiotic costs to the producer and supporting judicious antimicrobial use.

## INTRODUCTION

Bovine respiratory disease (BRD) is a disease complex encompassing animal, environment, and pathogen-related factors (Apley, 2006; Taylor *et al.*, 2010a). Due to numerous stress-related events, the incidence of BRD in the beef industry increases after weaning and impacts profitability due to elevated treatment costs, reduced weight gain, reduced feed efficiency, negative carcass outcomes, and increased mortality (Gardner *et al.*, 1999; Nickell *et al.*, 2008; Van Donkersgoed *et al.*, 2008a). Numerous management practices are put in place within all segments of the beef industry to reduce the risk of BRD and its negative downstream impact (Taylor *et al.*, 2010b). One specific practice, BRD control (i.e., metaphylaxis) is a common management practice that reflects the administration of an antibiotic to all cattle within a targeted group identified as being of elevated risk of developing BRD (Nickell and White, 2010). These populations are visibly healthy but contain individual animals possibly experiencing subclinical disease as well as healthy animals with a high probability of downstream disease development. The decision to administer an antibiotic labeled for BRD control to a population of calves is dependent upon exposure to risk factors (e.g., procurement location(s), body weight [BW], travel distance, weather events/variation, unknown prior vaccination status, and farms of origin) previously shown to be associated with an increased risk of developing BRD during the feeding period (Babcock et al., 2010, 2013a; Cernicchiaro et al., 2012a, 2012b, 2012c).

The current methodology of BRD control (i.e., administration of an antibiotic to 100% of calves within a targeted cohort) is a highly efficacious management practice repeatedly shown to reduce the negative impact of BRD among populations with exposure to known risk factors (Nickell and White, 2010). Numerous clinical trials reflecting decades of study data were recently compiled by meta-analysis and reported that injectable antibiotics (labeled for BRD control therapy) reduce the risk of BRD morbidity 1.5-fold on average compared to the placebo (O’Connor *et al.*, 2019). These outcomes subsequently are translated into economic value for the producer. Dennis *et al.* (2018) recently estimated that the practice of BRD control generates a net return of at least $532 million dollars to the U.S. beef industry.

Although efficacious, current BRD control practices involve the administration of an antibiotic to all individual calves within a defined group due to the inability to visually discern which individual calves may or may not benefit from BRD control therapy. Dedonder and Apley (2015) estimated that five calves must receive BRD control therapy in order to prevent one acute case of BRD. These results suggest, on average, that conventional BRD control antibiotics may be applied in excess to incoming cohorts of calves. The use of antimicrobials in bovine production can be costly to the producer and that cost is amplified (and value subsequently lost) when administered to animals without the possibility of value in return. Additionally, antibiotic use in animal agriculture is increasingly under pressure by human healthcare entities and regulatory bodies (Baptiste and Kyvsgaard, 2017; FDA, 2018; CDC, 2019). Prior efforts to reduce the economic impact of BRD control drug use have focused on the administration of the antibiotic to animals that display a rectal temperature exceeding a specific threshold (i.e., “temp and treat”). In contrast to traditional BRD control programs, this approach is designed to “diagnose” BRD at the specific time the animal is passing through the chute. However, prior research has shown a “temp and treat” program to be inferior to a conventional BRD control program (Vogel *et al.*, 1998). In addition, forgoing BRD control therapy to animals that could benefit from its application raises animal welfare concerns among veterinarians (Dean, 2011).

In contrast to the above issues, the ideal scenario would be to maintain BRD control antibiotic application but evolve the practice from group application to one that targets only individual animals at elevated risk for developing BRD. This scenario may optimize the BRD control practice for the producer and provide objective justification for this BRD management practice to the end consumer.

The Whisper On Arrival system is a chute-side technology developed to predict the risk of BRD manifestation among individual calves at the time of arrival to a backgrounding, stocker, or feedlot production system. The technology leverages four data points: heart sounds, lung sounds, body temperature, and BW. The heart and lung data are collected by a patented sound-collection device that is placed (by the user) on the right side of the calf’s thorax just caudal to the right humero-radial joint. Those data are wirelessly transmitted to the chute-side software application. The calf’s rectal temperature and BW are currently hand-entered into the software application. Those data are then analyzed by a proprietary machine-learning algorithm to provide the user with a “Treat” (individual calves that are to receive BRD control therapy) or “Do Not Treat” (individual calves that are left untreated) outcome.

The objective of this study was to compare health and performance outcomes among feedlot cattle managed by the following BRD control strategies: a targeted BRD control program based on individualized risk prediction generated by the Whisper On Arrival technology but at two different algorithm thresholds including 1) a conservative threshold targeted to reduce BRD control drug administration (compared to the positive control) yet administer BRD control therapy to the majority of animals and 2) an aggressive threshold designed to further reduce BRD control drug administration, 3) No BRD control drug administration (i.e., a negative control; 0% application to the targeted population) and 4) a conventional BRD control drug practice (i.e., a positive control; 100% application to the targeted population).

## MATERIALS AND METHODS

This multisite study was a joint effort among Merck Animal Health, Cactus Research (CR), Agri-Research Center (ARC), Bos Technica Research Services, Inc. (BT), and Midwest Veterinary Services, Inc. (MVS). Institutional Animal Care and Use Committee (IACUC) approval was not obtained at CR, ARC, and BT. However, IACUC approval was obtained by the researchers at the MVS study site. However, all sites followed the guidelines stated in the Guide for the Care and Use of Agricultural Animals in Agricultural Research and Teaching (FASS, 2010).

### Sample Population Description

Four separate clinical trials were executed at four independent sites. Study locations consisted of two sites in Texas (TX-1 and TX-2), one site in Oklahoma (OK), and one site in Nebraska (NE). At each of the four sites, the same study protocol was utilized that reflected the same four treatment groups incorporated into a completely randomized block design.

Across all four sites, the targeted sample population included auction market-derived steer calf lots considered by each site to be “medium” or “high-risk” for development of BRD based upon risk factors previously identified to be associated with an increased risk of BRD development (Cernicchiaro et al., 2012a, 2012b, 2012c; Babcock *et al.*, 2013a). Upon meeting these criteria, cattle were procured by all sites through standard industry channels. At arrival, only calves with a BRD clinical illness score (CIS) of ≤ 1 (Table 1) were eligible for study enrollment (Perino and Apley, 1998). Conversely, calves displaying BRD CISs of ≥ 2 or clinical signs of other infectious/noninfectious syndromes were not eligible for study enrollment.

**Table 1.** A description of CIS categories used across each study site to determine enrollment status and contribute to part of the BRD case definition

CIS	Observed behavior
0	• Bright, alert, responsive • No abnormal clinical signs
1	• Noticeable depression • May stand isolated with head down, ears drooping, but responsive to stimulation.
2	• Moderate depression • May remain recumbent or stand isolated with head down, depression obvious when stimulated • May stumble if forced to trot • Noticeable dyspnea with gauntness and nasal/ocular discharges
3	• Severe depression • Head carried low with ears drooping. Eyes dull, possible excess salivation/lacrimation • Pronounced dyspnea and gauntness. Mouth breathing. Nasal and ocular discharges
4	• Moribund and able to rise

### On-Arrival Processing, Randomization, and Animal Monitoring

Within 72 h post-arrival, each respective lot was administered a processing regimen consisting of the following products: a modified-live viral vaccine (Vista Once SQ; Merck Animal Health, Madison, NJ; 2 mL per animal), a multivalent clostridium vaccine (Vision 8; Merck Animal Health; 2 mL per animal), and an oral deworming agent (Safe-Guard suspension 10%, Merck Animal Health; 5 mg/kg BW). Each calf received an ear tag with a unique visual identification number.

In addition to the above products, each calf received a steroid implant that reflected the duration of animal ownership at each site. Calves at TX-1 were administered a 36 mg Zeranol implant at arrival (Ralgro, Merck Animal Health). Calves at TX-2 were administered an 80 mg trenbolone acetate (TBA) and 16 mg estradiol (E_2_) implant at arrival (Revalor IS, Merck Animal Health) and were reimplanted on Day 80 with a 200 mg TBA and 20 mg E_2_ implant (Revalor-200, Merck Animal Health). Calves at OK received a 200 mg TBA and 40 mg E2 implant at arrival (Revalor XS, Merck Animal Health). Calves enrolled at the MVS site received a 200 mg TBA and 40 mg E2 implant at arrival (Revalor XS, Merck Animal Health). At each site, implants were administered subcutaneously in the posterior aspect of the respective animal’s ear.

At each site, an 8 s sound consisting of individual heart and lung sounds were collected by a computer-aided auscultation system (Whisper Veterinary Stethoscope; DeDonder, 2010; Zeineldin, 2016; Baruch *et al.*, 2019). Sounds were collected on the cranio-ventral side of the right thorax just caudal to the calf’s humeroradial joint. In addition to lung and heart sounds, body temperature (obtained via rectal thermometer) and BW data were collected on each calf. The composited data were then analyzed by the Whisper On Arrival software. At each site, both the rectal thermometer and chute scale were certified to have been calibrated within 12 mo prior to study initiation.

The randomization schedule was generated in a commercial spreadsheet program (Microsoft Excel, Microsoft, Seattle, WA). While in the chute, each consecutive sequence of four calves were randomly allocated to one of four treatment groups: 1) negative control, 2) positive control (tildipirosin [TIL] to all animals), 3) Whisper-high (TIL only administered to cattle with BRD prediction probabilities above the predetermined threshold for this group), and 4) Whisper-low (TIL only administered to cattle with BRD prediction probabilities above the predetermined threshold for this group).

### Whisper On Arrival Overview

The Whisper On Arrival machine-learning algorithm was developed through a prior multisite study (i.e., algorithm development study) in which the above data (i.e., heart sounds, lung sounds, rectal temperature, and BW) were captured on all incoming cattle without the application of metaphylaxis. Cattle were followed to closeout and all health, performance, and carcass metrics were used to develop the algorithm tested in the current study (data not shown). For each individual animal, the algorithm generates a probability estimate of developing BRD. Established thresholds then allow for dictating when BRD control therapy is administered (i.e., a probability estimate above the threshold) or withheld (i.e., a probability estimate below the threshold). Therefore, the volume of animals that receive or do not receive BRD control therapy reflects where the threshold lies within the population’s BRD probability distribution.

Among the four treatment groups in the current study, two groups were managed by the Whisper On Arrival system. It was presumed that backgrounders and feedlot producers possess different levels of risk tolerance in forgoing BRD control therapy to proportions of cattle among incoming populations. Therefore, two proprietary thresholds consisting of a conservative (Whisper-high; high diagnostic sensitivity/low diagnostic specificity) and liberal (Whisper-low; low diagnostic sensitivity/high diagnostic specificity) threshold were selected a priori study initiation based on the algorithm generated in the algorithm development study (data not shown). These two thresholds were kept constant across all four study sites.

### Management of Study Population

All calves were penned by treatment group in open-air, dirt-floor pens whose size varied by study site (TX-2 and NE used 10 hd pens, TX-1 and OK used 70 hd pens). Calves allocated to either Whisper On Arrival-managed group were administered TIL (Zuprevo, Merck Animal Health; 4 mg/kg BW) for BRD control therapy based solely on the algorithm prediction (“Treat” or “Do Not Treat”). Therefore, calves allocated to either of these two treatment groups were housed in pens containing calves that were and were not administered TIL for BRD control.

At all sites, a 3-d post-metaphylactic interval was observed for all treatment groups. Beginning on d 3, all calves were eligible for BRD diagnosis and treatment. Daily observations were performed by trained study personnel masked to treatment group assignments. The BRD case definition across all sites reflected one of two profiles: 1) a CIS of 1 and a rectal temperature ≥40°C or 2) a CIS of 2 or 3 regardless of rectal temperature. Calves observed with a CIS of 4 were euthanized (Table 1). All calves were eligible for BRD treatment up to three times after metaphylaxis administration. The first BRD treatment consisted of florfenicol and flunixin meglumine (Resflor Gold; Merck Animal health; 40 mg florfenicol/kg and 2.2 mg flunixin/kg; subcutaneous [SC] administration). Following a 3-d posttreatment interval, cattle meeting the BRD case definition a second time received enrofloxacin (Baytril 100; Bayer Animal Health, Shawnee, KS; 12.5 mg/kg; SC). Following a 3-d posttreatment interval, if the case definition was met a third time, affected calves were administered oxytetracycline (Biomycin 200; Boehringer Ingelheim Animal Health, Duluth, GA; 9 mg/kg; SC). If more than three rounds of BRD therapy was found to be necessary, the calf was removed from the study population, a BW was captured, and treatment was administered, accordingly. However, the calf was considered off-study and all data were collected up to the day of removal. Similarly, each dead calf was individually weighed prior to necropsy and all data were collected up to the day of death.

### Nutritional Programs

At site TX-1, the same ration was fed for the duration of the 50-d study period. The ration was composed of 44.9% flaked corn, 7.3% corn dried-distillers grain, 17.3% corn wet distillers grain, 18.1% sweet bran, 7.1% corn stalks, and 5.3% supplement on a dry-matter basis. The ration contained Monensin (Rumensin; Zoetis, Kalamazoo, MI; 390 mg/hd/d) and Tylosin (Tylan 100; Elanco, Greenfield, IN; 90 mg/hd/d).

At site TX-2, calves were transitioned across three diets over a 61-d period. The finish ration consisted of 74.5% flaked corn, 8.5% alfalfa hay, 5.5% corn dried-distillers grains, 4% molasses blend, 2% fat, and 5.5% supplement on a DM basis. The finish ration contained Monensin (Rumensin; Zoetis, Kalamazoo, MI; 335 mg/hd/d) and Tylosin (Tylan; Elanco, Greenfield, IN; 100 mg/hd/d).

At site OK, cattle were transitioned across four diets over a 29-d period. The finish ration consisted of 85.9% flaked corn, 3.1% chopped alfalfa, 1.9% sorghum silage, 2.9% choice white grease, and 6.2% supplement on a DM basis. The finish ration contained Monensin (Rumensin; Zoetis, Kalamazoo, MI; 335 mg/hd/d) and Tylosin (Tylan 100; Elanco, Greenfield, IN; 90 mg/hd/d). Ractopamine (Optaflexx 45; Elanco, Greenfield, IN; 300 mg/hd) was included in the finish ration during the final 29 d on feed.

At site NE, cattle were transitioned across five diets over a 42-d period. The finish ration consisted of 57.4% high-moisture corn, 19.5% Modified distiller’s grains, 19.4% sweat bran, and 3.7% corn stalks on a dry-matter basis. This ration contained Monensin (Rumensin; Zoetis, Kalamazoo, MI; 390 mg/hd/d) and Tylosin (Tylan 100; Elanco, Greenfield, IN; 70 mg/hd/d).

At study conclusion (Days 50 and 60, sites TX-1 and NE, respectively; closeout, sites TX-2 [230 d] and OK [240 d]), individual BWs were captured on all calves still enrolled at that time. Health outcomes were captured at a short-term timepoint (50 or 60 d; all sites) and at closeout (sites TX-2 and OK). Performance outcomes were captured at a short-term timepoint (sites TX-1 and NE) and at closeout (TX-2 and OK). Carcass outcomes were captured for sites TX-2 and OK.

### Statistical Methods

Due to differences in cattle, feedlot management, pen sizes, and days on feed, data from the four different study sites were analyzed separately. The pen was considered the experimental unit and an alpha of ≤ 0.05 was considered significant. Raw data provided in spreadsheets were formatted for analysis and descriptive statistics were performed. Analyses were performed using linear mixed models (LMM) for a pen-level randomized complete block study design. Models were fitted using binomial (pen-level proportion outcomes), multinomial (ordinal carcass grades) or normal distributions (continuous outcomes), Kenward–Roger degrees of freedom, and Newton–Raphson and Ridging optimization procedures (Proc GLIMMIX SAS 9.4). Before making inferences from non-normal models, overdispersion was evaluated using Pearson chi-square/degrees of freedom (Laplace estimation method). A random intercept term was included in all models to account for the design structure (lack of independence among pens within blocks). Treatment group, coded as four categories, was included as a fixed effect.

## RESULTS

Across the four sites, 5,120 steer calves were allocated to their respective treatment groups. At the TX-1 site, 7 pens of 70 calves were allocated to each of the four treatment groups, originated from 4 different auction markets, were on study for 50 d, and averaged (range) 286.2 kg (173.3–440 kg) at arrival. At the TX-2 site, 20 pens of 10 calves were allocated to each of the four treatment groups, originated from 2 different auction markets, were on study until closeout (230 d) and averaged (range) 262.6 kg (209.6–435 kg) at arrival. At the OK site, 7 pens of 70 calves were allocated to each of the four treatment groups, originated from 3 different auction markets, were on study until closeout (240 d) and averaged (range) 278.1 kg (180.5–429.1 kg). At the NE site, 10 pens of 10 calves were allocated to each of the four treatment groups, originated from 2 different auction markets, were on study for 60 d and averaged (range) 236.3 kg (172.4–327.5 kg). Across all sites, no calves were removed between the time of removal to the time of processing for BRD or non-BRD syndromes.

Across all four sites, the number of cattle administered BRD control therapy was cumulatively reduced by 11% to 43% between the positive control (100% antibiotic application) and the two Whisper-managed treatment groups. Upon comparing each Whisper-managed group to the positive control, BRD control antibiotic use was reduced by 11% to 18% and 27% to 43% for the Whisper-high and Whisper-low groups, respectively. These outcomes are further displayed (by study site) in Table 2 and are reiterated in Tables 3–7.

**Table 2.** Proportion of BRD control antibiotic application among all treatment groups at each of the four study sites

	Study site			
Treatment group	TX-1	TX-2	OK	NE
Negative control	0%	0%	0%	0%
Positive control	100%	100%	100%	100%
Whisper-high	89%	82%	87%	82%
Whisper-low	73%	70%	63%	57%

TIL was administered to all calves in the positive control group and only to calves identified as being at risk for developing BRD in both Whisper-managed groups.

**Table 3.** Model-adjusted means and standard errors of the means (SEM) for the short-term (50–60 d on feed) health outcomes among each of the four study sites*

Outcomes	Negative control		Positive control		Whisper-high		Whisper-low		*P*-value†
	Mean	SEM	Mean	SEM	Mean	SEM	Mean	SEM	
TX-1									
BRD control drug, %	0%		100%		89.3%		72.7%		
Days on feed	50		50		50		50		
In weight, kg	288.9	7.3	284.9	7.3	283.5	7.3	288.5	7.3	0.43
BRD morbidity	18.9%^a^	3.4%	5.9%^b^	1.5%	7.6%^b^	1.8%	9.2%^b^	2.0%	<0.01
BRD 2nd treatments	3.3%^a^	1.4%	0.7%^b^	0.4%	0.6%^b^	0.6%	1.2%^b^	0.6%	<0.01
BRD 3rd treatments	0.7%	0.4%	0.6%	0.4%	0.7%	0.4%	0.6%	0.4%	0.96
BRD case-fatality	3.1%	1.8%	9.7%	5.3%	7.5%	4.2%	6.3%	3.5%	0.54
BRD mortality	0.5%	0.4%	0.9%	0.5%	0.5%	0.4%	0.5%	0.4%	0.82
Overall mortality	0.6%	0.4%	1.1%	0.6%	0.6%	0.5%	0.9%	0.4%	0.64
TX-2									
BRD control drug, %	0%		100%		82%		69.5%		
Days on feed	60		60		60		60		
In weight, kg	263.5	2.3	260.8	2.3	261.7	2.3	261.7	2.3	0.33
BRD morbidity	27.7%^a^	5.5%	15.9%^b^	3.9%	13.4%^b^	3.5%	16.4%^b^	4.0%	<0.01
BRD 2nd treatments	6.6%	3.2%	2.4%	1.4%	2.8%	1.6%	4.3%	2.2%	0.08
BRD 3rd treatments	2.6%	1.5%	1.1%	0.8%	0.4%	0.4%	2.6%	1.5%	0.17
BRD case-fatality	3.6%	2.5%	6.1%	4.2%	0.0%	0.0%	8.8%	4.9%	0.79
BRD mortality	1.0%	0.7%	1.0%	0.7%	0.0%	0.0%	1.5%	0.9%	0.96
Overall mortality	1.0%	0.7%	1.0%	0.7%	0.0%	0.0%	1.5%	0.9%	0.96
OK									
BRD control drug, %	0%		100%		87.2%		62.9%		
Days on feed	60		60		60		60		
In weight, kg	277.6	12.7	278.5	12.7	278.1	12.7	277.6	12.7	0.89
BRD morbidity	17.5%^a^	7.5%	8.6%^b^	4.2%	10.3%^b^	4.8%	10.2%^b^	4.9%	<0.01
BRD 2nd treatments	4.9%^a^	2.2%	2.2%^b^	1.1%	2.6%^b^	1.3%	4.0%^b^	1.9%	0.04
BRD 3rd treatments	3.2%^a^	1.2%	1.1%^b^	0.5%	1.2%^b^	0.6%	2.7%^a, b^	1.0%	0.04
BRD case-fatality	1.7%	1.2%	0.0%	0.0%	1.4%	71.0%	4.1%	2.3%	0.71
BRD mortality	0.6%	0.4%	0.0%	0.0%	0.2%	68.0%	0.8%	0.4%	0.68
Overall mortality	0.6%	0.4%	0.0%	0.0%	0.6%	98.0%	0.8%	0.4%	0.98
NE									
BRD control drug, %	0%		100%		82%		57%		
Days on feed	60		60		60		60		
In weight, kg	234.5	2.1	235.9	2.1	238.1	2.1	235.9	2.1	0.75
BRD morbidity	34.6%	15.8%	36.8%	16.2%	44.5%	17.2%	44.5%	17.2%	0.38
BRD 2nd treatments	20.4%	10.6%	15.5%	8.7%	24.4%	11.9%	26.5%	12.5%	0.24
BRD 3rd treatments	16.0%	3.7%	11.0%	3.1%	18.0%	3.8%	17.0%	3.8%	0.54
BRD case-fatality	0.0%	0.0%	5.3%	3.6%	2.2%	2.2%	0.0%	0.0%	0.91
BRD mortality	0.0%	0.0%	3.0%	1.7%	1.0%	1.0%	0.0%	0.0%	0.82
Overall mortality	0.0%	0.0%	3.0%	1.7%	1.0%	1.0%	0.0%	0.0%	0.82

*Within each site, the percentage (%) of steers administered BRD control therapy is noted below each respective treatment group. Mixed models with a random effect to account for lack of independence among pens within blocks.

†When overall *P*-values are ≤ 0.05, means with different superscripts within rows differ significantly (*P* ≤ 0.05). Not adjusted for multiple comparisons.

**Table 4.** Model-adjusted means for the short-term (50–60 d on feed) performance outcomes among two of the four study sites (TX-1 and NE)*

Outcomes	Negative control		Positive control		Whisper-high		Whisper-low		*P*-value†
	Mean	SEM	Mean	SEM	Mean	SEM	Mean	SEM	
TX-1									
BRD control drug application, %	0%		100%		89.3%		72.7%		
Days on feed	50		50		50		50		
Average in weight, kg	288.5	5.9	288.5	5.9	286.2	5.9	291.2	5.9	0.22
Average final weight, kg	332.5^a^	8.5	342.5^b^	8.5	338.8^a,b^	8.5	343.4^b^	8.5	<0.01
ADG, deads-out; kg/d‡	0.9^a^	0.1	1.1^b^	0.1	1.1^b^	0.1	1.1^b^	0.1	<0.01
ADG, deads-in; kg/d	0.8^a^	0.1	1.0^b^	0.1	1.0^b^	0.1	1.0^b^	0.1	0.01
Mean daily DMI, kg/d	6.6^a^	0.2	7.0^b^	0.2	7.0^b^	0.2	7.0^b^	0.2	<0.01
G:F, deads-out	0.13^a^	0.01	0.16^b^	0.01	0.1524^b^	0.01	0.1498^a,b^	0.01	<0.01
G:F, deads-in	0.13	0.01	0.15	0.01	0.15	0.01	0.14	0.01	0.19
NE									
BRD control drug application, %	0%		100%		82%		57%		
Days on feed	60		60		60		60		
Average in weight, kg	234.5	2.3	235.9	2.3	238.1	2.3	235.9	2.3	0.75
Average final weight, kg	321.6	7.7	324.8	7.7	325.7	7.7	319.8	7.7	0.53
ADG, deads-out; kg/d	1.4	0.1	1.5	0.1	1.4	0.1	1.4	0.1	0.80
ADG, deads-in; kg/d	0.4	0.5	0.5	0.5	0.2	0.5	0.4	0.5	0.70
Mean daily DMI, kg/d	7.0	0.6	7.1	0.6	6.9	0.6	7.2	0.6	0.50
G:F, deads-out	0.20	0.03	0.21	0.03	0.21	0.03	0.21	0.03	0.81
G:F, deads-in	0.07	0.08	0.08	0.08	0.03	0.08	0.06	0.08	0.87

*Within each site, the percentage (%) of steers administered BRD control therapy is noted below each respective treatment group. Mixed models with a random effect to account for lack of independence among pens within blocks.

†When overall *P*-values are ≤ 0.05, means with different superscripts within rows differ significantly (*P* ≤ 0.05). Not adjusted for multiple comparisons.

‡Deads-in/deads-out calculations reflect the inclusion (deads-in) or exclusion (deads-out) of cattle that died during the study duration.

**Table 5.** Model-adjusted means for the closeout health outcomes among two of the four study sites (TX-2 and OK)

Outcomes	Negative control		Positive control		Whisper-high		Whisper-low		
	Mean	SEM	Mean	SEM	Mean	SEM	Mean	SEM	*P*-value†
TX-2									
BRD control drug application, %	0%		100%		82%		69.5%		
Day on feed	233		233		233		233		
BRD morbidity	28.3%^a^	5.5%	17.5%^b^	4.1%	15.0%^b^	3.7%	18.0%^b^	4.2%	<0.01
BRD 2nd treatments	7.9%	3.2%	4.5%	2.0%	4.0%	1.9%	6.2%	2.6%	0.26
BRD 3rd treatments	2.9%	1.7%	1.4%	1.0%	0.7%	0.6%	2.9%	1.7%	0.20
BRD case-fatality	5.3%	3.0%	5.6%	3.8%	6.5%	4.4%	10.8%	5.1%	0.75
BRD mortality	1.5%	0.9%	1.0%	0.7%	1.0%	0.7%	2.5%	1.1%	0.70
Overall mortality	1.9%	1.1%	1.5%	0.9%	1.9%	1.1%	3.4%	1.5%	0.57
OK									
BRD control drug application, %	0%		100%		87.2%		62.9%		
Day on feed	240		240		240		240		
BRD morbidity	22.0%^a^	6.27%	11.5%^b^	3.8%	13.2%^b^	4.3%	13.4%^b^	4.3%	<0.01
BRD 2nd treatments	6.4%	2.2%	3.5%	1.3%	3.5%	1.3%	5.0%	1.8%	0.06
BRD 3rd treatments	4.2%^a^	1.3%	1,8%^b^	0.7%	1.6%^b^	0.7%	3.3%^b^	1.1%	0.04
BRD case-fatality	3.2%	1.6%	1.5%	1.4%	1.3%	1.3%	6.3%	2.7%	0.33
BRD mortality	1.2%	0.5%	0.2%	0.2%	0.4%	0.3%	1.2%	0.5%	0.20
Overall mortality	1.8%	0.6%	1.0%	0.5%	1.2%	0.5%	1.8%	0.6%	0.63

*Mixed models with a random effect to account for lack of independence among pens within blocks.

†When overall *P*-values are ≤ 0.05, means with different superscripts within rows differ significantly (*P* ≤ 0.05). Not adjusted for multiple comparisons.

**Table 6.** Model-adjusted means for the closeout performance outcomes among two of the four study sites (TX-2 and OK)*

Outcomes	Negative control		Positive control		Whisper-high		Whisper-low		
	Mean	SEM	Mean	SEM	Mean	SEM	Mean	SEM	*P*-value†
TX-2									
BRD control drug application, %	0%		100%		82%		69.5%		
Day on feed	233		233		233		233		
Average in weight, kg	263.5	2.3	260.8	2.3	261.7	2.3	261.7	2.3	0.33
Average final weight, kg	597.4	7.2	601.5	7.2	611.9	7.2	597.8	7.2	0.13
ADG, deads-out; kg/d‡	1.5	0.0	1.5	0.0	1.5	0.0	1.5	0.0	0.65
ADG, deads-in	1.4	0.0	1.5	0.0	1.4	0.0	1.5	0.0	0.45
Mean daily DMI	8.1	0.1	8.3	0.1	8.4	0.1	8.1	0.1	0.18
G:F, deads-out	0.18	0.003	0.18	0.003	0.18	0.003	0.18	0.003	0.62
G:F, deads-in	0.17	0.003	0.17	0.003	0.17	0.003	0.17	0.003	0.94
OK									
BRD control drug application, %	0%		100%		87.2%		62.9%		
Days on feed	240		240		240		240		
Average in weight, lbs	281.8	12.7	282.3	12.7	280.9	12.7	281.4	12.7	0.51
Average final weight, pen; lbs	631.4	8.2	640.9	8.2	640.0	8.2	642.7	8.2	0.13
ADG, deads-out; kg/d	1.5	0.1	1.5	0.1	1.5	0.1	1.5	0.1	0.17
ADG, deads-in; kg/d	1.36^a^	0.05	1.45^b^	0.05	1.46^b^	0.05	1.43^a,b^	0.05	0.05
Mean daily DMI	8.3	0.2	8.3	0.2	8.6	0.2	8.4	0.2	0.06
G:F, deads-out	0.18	0.002	0.18	0.002	0.18	0.002	0.18	0.002	0.18
G:F, deads-in	0.16	0.003	0.17	0.003	0.17	0.003	0.17	0.003	0.16

*A 4% shrink was applied to closeout weights. Mixed models with a random effect to account for lack of independence among pens within blocks.

†When overall *P*-values are ≤ 0.05, means with different superscripts within rows differ significantly (*P* ≤ 0.05). Not adjusted for multiple comparisons.

‡Deads-in/deads-out calculations reflect the inclusion (deads-in) or exclusion (deads-out) of cattle that died during the study duration.

**Table 7.** Model-adjusted means for the carcass outcomes among two of the four study sites (TX-2 and OK)*

Outcomes		Negative control		Positive control		Whisper-high		Whisper-low		*P*-value†
		Mean	SEM	Mean	SEM	Mean	SEM	Mean	SEM	
TX-2										
BRD control drug application, %		0%		100%		82%		69.5%		
Hot carcass weight, kg		376.4	4.0	380.9	4.0	385.5	4.0	380.0	4.0	0.45
Yield, %		60.8	0.3	61.3	0.3	61.2	0.3	61.3	0.3	0.54
Ribeye area		14.7	0.2	14.3	0.2	14.6	0.2	14.4	0.2	0.31
Marbling		475	7.6	486	7.6	482	7.6	473	7.6	0.57
Backfat		Not reported (NR)	NR	NR	NR	NR	NR	NR	NR	-
Calculated yield grade		2.8	0.1	2.9	0.1	2.8	0.1	2.8	0.1	0.82
Yield grade	% of treatment group (count)									0.21
	1	9.9% (13)		9.7% (13)		14.0% (19)		10.4% (14)		*N* = 536‡
	2	59.5% (78)		45.5% (61)		44.1% (60)		46.7% (63)		
	3	23.7% (31)		34.3% (46)		36.0% (49)		35.6% (48)		
	4	6.9% (9)		8.2% (11)		4.4% (6)		7.4% (10)		
	5	0.0% (0)		2.2% (3)		1.5% (2)		0.0% (0)		
Quality grade	% of treatment group (count)									0.98
	Prime	5.3% (7)		5.2% (7)		1.5% (2)		1.5% (2)		*N* = 537
	Choice	76.3% (100)		76.3% (103)		82.4% (112)		84.4% (114)		
	Select	18.3% (24)		18.5% (25)		15.4% (21)		13.3% (18)		
	Other	0.0% (0)		0.0% (0)		0.7% (1)		0.7% (1)		
OK										
BRD control drug application, %		0%		100%		87.2%		62.9%		
Hot carcass weight, kg		410.0^a^	3.5	415.5^b^	3.5	417.8^b^	3.5	411.4^a^	3.5	0.03
Yield, %		65.2	0.4	65.0	0.4	65.4	0.4	64.3	0.4	0.14
Ribeye area		14.8	0.2	14.8	0.2	15.0	0.2	14.6	0.2	0.08
Marbling		515	10.3	502	10.3	502	10.3	503	10.3	0.31
Backfat		0.7	0.0	0.7	0.0	0.7	0.0	0.7	0.0	0.76
Calculated yield grade		3.2	0.1	3.3	0.1	3.2	0.1	3.3	0.1	0.50
Yield grade	% of treatment group (count)									0.38
	1	8.5% (39)		9.2% (44)		15.2% (72)		7.3% (34)		*N* = 1,880
	2	34.4% (158)		30.5% (146)		30.8% (146)		32.6% (152)		
	3	37.0% (170)		38.8% (186)		35.2% (167)		36.8% (172)		
	4	17.8% (82)		18.8% (90)		15.8% (75)		19.5% (91)		
	5	2.4% (11)		2.7% (13)		3.0% (14)		3.9% (18)		
Quality grade	% of treatment group (count)									0.44
	Prime	6.1% (28)		4.6% (22)		16.7% (79)		4.5% (21)		*N* = 1,879
	Choice	82.6% (380)		84.6% (405)		74.6% (353)		86.9% (406)		
	Select	10.7% (49)		10.9% (52)		8.5% (40)		8.6% (40)		
	Other	0.7% (3)		0.0% (0)		0.2% (1)		0.0% (0)		

*Mixed models with a random effect to account for lack of independence among pens within blocks.

†When overall *P*-values are ≤ 0.05, means with different superscripts within rows differ significantly (*P* ≤ 0.05). Not adjusted for multiple comparisons.

‡Final carcass counts reflected in the analysis are less than actual enrollment numbers due to inability to retrieve data from all lots.

Health (short-term duration and closeout timepoints), live performance (short-term duration and closeout timepoints), and carcass outcomes are displayed in Tables 3–7, respectively. Regarding health outcomes at the short-term timepoint (measured at all 4 sites), the positive control and both Whisper-managed groups displayed an improvement (*P* ≤ 0.05) in BRD morbidity at three sites (TX-1, TX-2, and OK) but not site NE, BRD 2nd treatments at sites TX-1 and OK but not sites TX-2 and NE, and BRD 3rd treatments at site OK but not sites TX-1, TX-2, and OK compared to the negative control (Table 3). Likewise, regarding performance outcomes measured at the short-term timepoint (sites TX-1 and NE), the positive control and both Whisper-managed groups displayed an improvement (*P* ≤ 0.05) in average final weight, average daily gain (ADG; deads-in/deads-out), dry-matter intake, and feed efficiency (deads-in/deads-out) compared to the negative control at site TX-1 but not site NE (Table 3). At closeout (measured at sites TX-2 and OK), the positive control and both Whisper-managed groups displayed an improvement (*P* ≤ 0.05) compared to the negative control in BRD morbidity (both sites) and BRD 3rd treatments at site OK but not site TX-2 (Table 5). Regarding closeout performance, the positive control and both Whisper-managed groups displayed an improvement (*P* ≤ 0.05) in ADG (deads-in) compared to the negative control at site OK (Table 6). Among carcass outcomes, the positive control and Whisper-high group hot-carcass weights were greater (*P* ≤ 0.05) than the negative control at site OK but not site TX-2 (Table 7). No further carcass characteristics were observed to be different (*P* > 0.05; Table 7). Among these respective outcomes at these sites, both Whisper-managed groups displayed no differences (*P* > 0.05) compared to the positive control. No differences (*P* > 0.05) were observed in BRD-related or overall mortality across all sites at either the short-term timepoints or at closeout. At the NE site, no differences (*P* > 0.05) in health or performance were observed across all treatment groups (Tables 3 and 4).

## DISCUSSION

The objective of this study was to compare health and performance outcomes among a targeted BRD control program based on individualized risk predictions generated by the Whisper On Arrival technology (at two different threshold settings; Whisper-high and Whisper-low), a negative control (i.e., no BRD control drug administration), and a positive control (i.e., conventional BRD control drug practices; 100% application to the targeted population). Across four study sites, the Whisper On Arrival technology reduced BRD control antibiotic use by 11% to 43% without evidence of negative impacts on health, performance, and carcass metrics as compared to the positive control (100% receiving the BRD control drug). Therefore, in this study, the Whisper On Arrival technology showed no difference compared to a conventional BRD control program. However, BRD control antibiotic costs to the producer were reduced across sites and supported judicious antimicrobial use.

Administering an antimicrobial labeled for BRD control is highly effective in reducing the negative effects of BRD (Nickell *et al.*, 2008; Van Donkersgoed *et al.*, 2008b; Nickell and White, 2010; Van Donkersgoed, 2013; Tennant *et al.*, 2014). A recent meta-analysis estimated that the administration of an injectable antibiotic for BRD control reduces the risk of BRD morbidity by 1.5-fold on average within the first 45 d on feed (O’Connor *et al.*, 2019). The decision to administer a BRD control antimicrobial to an incoming population of animals is based on known exposure to risk factors associated with BRD. However, exposure of calf cohorts to these BRD risk factors do not accurately predict BRD cumulative incidence at the group level (Babcock *et al.*, 2013b). This supports recent work that estimates only one in five animals observe a benefit (i.e., reduction in BRD morbidity risk) from BRD control therapy (DeDonder and Apley, 2015). These data indicate that evolving the conventional BRD control management practices to one that targets individuals at greater risk of developing BRD may provide value to stakeholders within the beef supply chain (e.g., producers, veterinarians, retailers, and consumers).

At each of the four study sites, the targeted enrollment population included cattle classified as being of medium to high risk of developing BRD. Given the challenges in using risk factor exposure as a means of predicting BRD incidence at the group level, there is an inherently large degree of variability in potential outcomes (Babcock *et al.*, 2013b). That said, among the negative control treatment groups across sites, BRD morbidity ranged from 18.9% (TX-1) to 34.6% (NE) at the short-term timepoint. However, BRD mortality ranged from 0% (NE) to 1% (TX-2) among the negative controls. Based on these health outcomes, although standardized definitions do not exist, one could argue that all four study populations reflect medium-risk populations rather than high-risk populations. Study populations from prior research designed to study medium/moderate-risk cattle parallel the health outcomes in this study further confirming this observation (Van Donkersgoed *et al.*, 2008b; Tennant *et al.*, 2014). More research is necessary to assess the efficacy of the technology among populations at a greater risk of developing BRD.

Although not unexpected, variability was observed in conventional BRD control efficacy (i.e., efficacy of the positive control compared to the negative control) across study sites. Prior efforts evaluating the body of BRD control literature have observed large ranges in BRD control drug efficacy (DeDonder and Apley, 2015; O’Connor *et al.*, 2019). That said, the traditional BRD control program implemented in this study has previously been shown to be effective (Intervet, 2012; Compiani, 2014; Teixeira *et al.*, 2017; Celestino *et al.*, 2020) and proved to be efficacious at three of the four sites. At one study site (NE), no significant differences were observed in any of the outcome variables compared to the positive control or with either of the Whisper-managed groups. Additionally, among the three sites in which efficacy was observed, the magnitude of the effect (i.e., the number of outcome variables in which statistical differences were identified) differed as well. Given these variances, it is likely prudent to consider the larger system that is being managed and the factors that may influence BRD outcomes. First and foremost, we understand that current BRD diagnostic modalities are significantly flawed (due to the subjective nature of clinical signs) and likely distort the true cumulative incidence of BRD morbidity (White and Renter, 2009; Timsit *et al.*, 2016). For example, the NE site observed the highest degree of morbidity within the negative control group (34.6%); however, virtually no mortality was observed during that 60-d study duration. Therefore, one possible explanation for the outlying outcomes at the NE site is that those respective study personnel may be more sensitive (and subsequently less specific) in their BRD detection efforts. Additional parameters that likely influence the magnitude of BRD include prepartum (Van Donkersgoed *et al.*, 1995; Van Eenennaam *et al.*, 2014), preweaning (Hanzlicek *et al.*, 2013; Murray *et al.*, 2016), weaning (Taylor *et al.*, 2020), and postweaning factors (Cernicchiaro et al., 2012a, 2012b, 2012c). Therefore, although there may be scenarios where drug efficacy is specifically associated with a lack of BRD reduction between the positive and control group, it is likely that there are additional factors at play that we are unable to currently measure and subsequently manage in a study design like that implemented in the current study. More research is necessary to determine if animal and/or cohort physiological profiles impact response to current BRD control measures.

Variability was observed across sites relative to the proportion of animals receiving BRD control therapy in both Whisper On Arrival-managed groups. Given likely differences in the magnitude of BRD risk factor exposure from cohort to cohort, it is probable that the actual BRD risk profile of each group will also vary. Therefore, it was not unexpected to observe differences in the proportion of animals receiving BRD control therapy, not only across sites, but also across different lots within a site (data not shown). In practice, the threshold setting for the Whisper On Arrival system is dictated by the user and may be modified based on their perceived risk classification of each incoming lot. However, more research is necessary to determine if the Whisper On Arrival threshold can be optimized based on the quantified magnitude of BRD risk factor exposure.

The original Whisper system (Whisper Veterinary Stethoscope) is applied exclusively to sick cattle to estimate lung health at the time of BRD diagnosis (DeDonder, 2010; Timsit *et al.*, 2016; Zeineldin, 2016; Baruch *et al.*, 2019). In contrast, the Whisper On Arrival technology predicts the likelihood of a future clinical BRD event (rather than confirm a diagnosis on a sick animal) and is applied to cattle at the time of arrival. Cattle at feedlot arrival, compared to the clinically ill population, reflect a population experiencing a different physiological and disease state (Hanzlicek *et al.*, 2010; Baruch *et al.*, 2019). Therefore, permutations of lung sound data and additional information (heart sound metrics, rectal temperature, and BW) were found to be necessary to optimize the robustness of the predicted estimate (data not shown). Additionally, in contrast to the Whisper Veterinary Stethoscope technology, the Whisper On Arrival system utilizes a novel sound capture device that is more conducive to the ergonomics and safety profile necessary for processing incoming cattle. Nonetheless, given that the Whisper On Arrival system represents an additional processing event, costs associated with additional labor, time, and cost of the technology must be evaluated by the producer.

Beef producers continuously search for cost management opportunities, while veterinarians strive to not only support their client’s economic endeavors but also sustain the tools (e.g., antibiotics) implemented to reduce disease pressure within production systems. Both entities are also widely cognizant of market factors that may affect producer demand. The Whisper On Arrival technology was developed to 1) optimize the cost associated with this practice by individually identifying cattle at risk of developing BRD, subsequently providing actionable data for the user leading to a decision to administer a BRD control antimicrobial or forgo therapy and 2) support judicious antimicrobial use. Across four study sites, the Whisper On Arrival technology reduced BRD control antibiotic use 11% to 43% without negative impacts on health, performance, and carcass metrics as compared to the positive control (100% receiving the BRD control drug). Therefore, the Whisper On Arrival technology maintained the benefits of a conventional BRD control program while reducing antibiotic costs to the producer and supporting judicious antimicrobial use.
